# Endothelial Dysfunction in COVID-19: Potential Mechanisms and Possible Therapeutic Options

**DOI:** 10.3390/life12101605

**Published:** 2022-10-14

**Authors:** Maria Chiara Pelle, Isabella Zaffina, Stefania Lucà, Valentina Forte, Vincenzo Trapanese, Melania Melina, Federica Giofrè, Franco Arturi

**Affiliations:** 1 Unit of Internal Medicine, Department of Medical and Surgical Sciences, University “Magna Graecia” of Catanzaro, 88100 Catanzaro, Italy; 2 Research Centre for the Prevention and Treatment of Metabolic Diseases (CR METDIS), University “Magna Graecia” of Catanzaro, 88100 Catanzaro, Italy

**Keywords:** COVID-19, SARS-CoV-2, inflammation, endothelium, pathogenesis, coagulopathy

## Abstract

SARS-CoV-2, a novel coronavirus found in Wuhan (China) at the end of 2019, is the etiological agent of the current pandemic that is a heterogeneous disease, named coronavirus disease 2019 (COVID-19). SARS-CoV-2 affects primarily the lungs, but it can induce multi-organ involvement such as acute myocardial injury, myocarditis, thromboembolic eventsandrenal failure. Hypertension, chronic kidney disease, diabetes mellitus and obesity increase the risk of severe complications of COVID-19. There is no certain explanation for this systemic COVID-19 involvement, but it could be related to endothelial dysfunction, due to direct (endothelial cells are infected by the virus) and indirect damage (systemic inflammation) factors. Angiotensin-converting enzyme 2 (ACE2), expressed in human endothelium, has a fundamental role in severe acute respiratory syndrome coronavirus 2 (SARS-CoV-2) infection. In fact, ACE2 is used as a receptor by SARS-CoV-2, leading to the downregulation of these receptors on endothelial cells; once inside, this virus reduces the integrity of endothelial tissue, with exposure of prothrombotic molecules, platelet adhesion, activation of coagulation cascades and, consequently, vascular damage. Systemic microangiopathy and thromboembolism can lead to multi-organ failure with an elevated risk of death. Considering the crucial role of the immunological response and endothelial damage in developing the severe form of COVID-19, in this review, we will attempt to clarify the underlying pathophysiological mechanisms.

## 1. Introduction

COVID-19 is caused by SARS-CoV-2 and may be characterized as a multisystem disease [1]. Indeed, although COVID-19 was initially considered to be a respiratory disease, it is associated with a number of extrapulmonary complications. Several macro-thromboembolic events (e.g., pulmonary embolism), micro-thromboembolic events (e.g., microthrombosis in small pulmonary arteries) and/or disseminated intravascular coagulation have been described in patients with COVID-19. To date, growing evidence supports the key role of endothelial dysfunction (ED) in the pathogenesis of COVID-19 and in determining its severity [2]. Endothelial cells have many functional properties and are key regulators of coagulation, oxidative stress, inflammation and vasomotricity. Viral diseases can damage the endothelium in many ways. Recent evidence suggests that COVID-19 can induce ED by both direct viral effects and virus-dependent activation of the inflammatory response [3]. Vascular damage is probably related both to the direct cytopathic effect of the virus on endothelial cells (ECs) and to high levels of cytokines and other inflammatory markers, inducing systemic endotheliitis, leucocyte adhesion, platelet activation and reduced nitric oxide (NO) bioavailability [3,4]. The activation of ECs induces localized inflammation and increases the production of reactive oxidative species and thrombotic disease not only in the pulmonary circulation but also in peripheral vessels [5]. Circulating ECs increase, as well as platelets, lymphocytes and the inflammatory endothelial marker soluble vascular cell adhesion molecule 1 (sVCAM1) [6,7]. Several studies have demonstrated that severe pulmonary manifestations in COVID-19 patients are not only due to acute respiratory distress syndrome (ARDS) but also to macro- and microvascular involvement, with vascular endothelial injury and subsequent dysfunction [8]. Similarly, numerous extrapulmonary symptoms have been related toendothelium damage [9,10]. Several sources of evidence suggest that the endothelium may represent an important target for novel treatments [11].

As clinical evidence indicates a crucial endothelial contribution to the clinical manifestations of COVID-19, the aim of this review is to summarize the pathophysiology of ED in this clinical setting, with a focus on pharmacological strategies targeting ED.

## 2. Tropism of SARS-CoV-2 for Endothelium

SARS-CoV-2 attacks the lung and other organs using the ACE2 receptor expressed in the lungs and different organs, explaining why COVID-19 can have different manifestations. Moreover, many studies have demonstrated that patients with risk factors, such as cardiovascular disease, hypertension, obesity and diabetes, share a common alteration: ED that appears to be induced directly by SARS-CoV-2 infection and indirectly by systemic inflammation. The RNA genome of SARS-CoV-2encodes about 29 proteins, among these, the noteworthy ones are spike (S), envelope (E), membrane-associated (M) and nucleocapsid (N) proteins. The S protein is composed of two subunits: S1 of the receptor-binding domain (RBD) that binds ACE2 and S2 that anchors the virion to the membrane leading to fusion [12]. Subunit S2 is activated thanks to the transmembrane protease serine protease-2 (TMPRSS-2) and ADAM metallopeptidase domain 17 (ADAM17) of the host cell [13]. The virus enters the host cell via endocytosis; viral RNA is released and is ready for replication of new virions [14].

**Figure 1:**
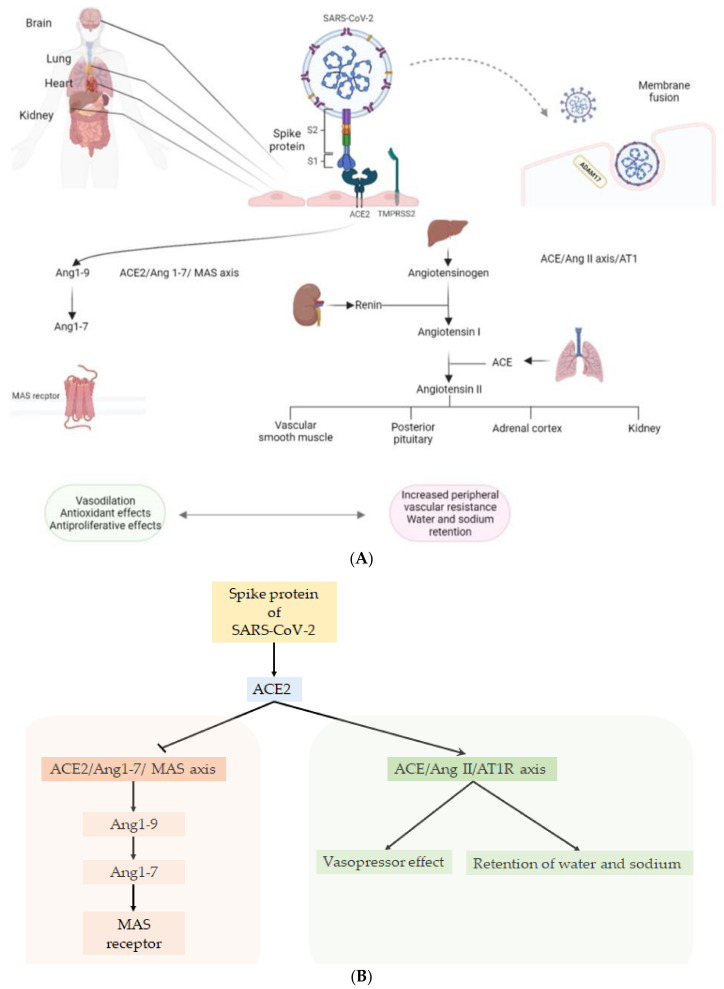
(**A**) The S protein of the virus attacks the lung and other organs, due to using the ACE2 receptor. The S protein is composed of two subunits: S1 of the RBD that binds ACE2 and S2 that anchors the virion to the membrane leading the fusion. Subunit S2 is activated thanks to the TMPRSS-2and ADAM17 of the host. In the lung, through the ACE/Ang II axis/AT1 system, ACE converts Ang I into Ang II, which leads to the release of aldosterone leading to a vasopressor effect, with increased peripheral vascular resistance and retention of water and sodium due to release of aldosterone. (**B**) The action of ACE2, on the other hand, generates several molecules (Ang (1–9), Ang (1–5) and Ang (1–7)); Ang (1–7) constitutes the final molecule in this series and acts on the Mas receptor, promoting vasodilation and antioxidant and antiproliferative effects. SARS-CoV-2 competes with Ang II for ACE2 and this leads to its downregulation, determining an imbalance of ACE/ACE2, and it hyperactivates the ACE/Ang II/AT1R axis, resulting in a decrease inAng (1–7) [12,13,14,15,16,17,18,19,20].

ACE2 is known, above all, as an enzyme and is important in the function of the renin–angiotensin–aldosterone system (RAAS). RAAS is a signaling pathway that controls blood pressure, systemic and local blood flow and natriuresis and includes renin, angiotensinogen and angiotensin (Ang) I and II and their AT1 receptor [15,16]. In the lung, ACE converts AngI into AngII, which leads to the release of aldosterone. This subsystem, called the ACE/Ang II axis/AT1 receptor, has a vasopressor effect, with increased peripheral vascular resistance and retention of water and sodium due to the release of aldosterone. The action of ACE2 generates a series of molecules with individual activities (Ang (1–9), Ang (1–5) and Ang (1–7)); Ang (1–7) constitutes the final molecule in this series and acts on the Mas receptor, and it promotes vasodilation, antioxidant and antiproliferative effects [17,18,19]. Activation of the ACE2/Ang (1–7)/Mas axis mitigates the effects of the ACE/Ang II/AT1R axis. SARS-CoV-2 competes with Ang II for ACE2 and leads to its downregulation. Virus binding determines an imbalance of ACE/ACE2 and it hyperactivates the ACE/Ang II/AT1R axis, leading to a decrease in Ang (1–7) [20] (Figure 1A,B).

Patients with chronic inflammatory states have an imbalance between classic and alternative RAAS; this leads to an increased risk of infection and a higher likelihood of developing complications [21].

COVID-19 causes alteration in the functions and permeability of ECs [22], although the exact mechanism is not known. Some studies suggest that the S protein is the cause of endothelial damage by interaction with integrin α5β1 and the nuclear factor κB (NF-κB) pathway [23]. Moreover, this virus seems capable of altering the integrity of the endothelial glycocalyx; in fact, the study by Stahl et al. found increased expression of the Tie-2 receptor and levels of syndecan-1 (SDC1), a heparin 152 sulfate proteoglycan, in the 151 serum and plasma samples from patients, which is a sign of disruption of the glycocalyx. Another study, conducted on autopsy findings, revealed viral inclusions in apoptotic ECs and infiltration of inflammatory cells around the vessels and ECs, consistent with endotheliitis [24].

These observations suggest a strong tropism of the virus to the endothelium and that its apoptosis promotes disruption of the endothelial barrier with interstitial edema and increased recruitment of circulating activated immune cells.

## 3. Endothelial Dysfunction: Hypercoagulability and Inflammation

The endothelium is not only a thin cell layer (0.2 μm thick) of all types of vessels that divide blood from tissue but a dynamic organ that is able to modify its status according to external conditions and stress [25].

### 3.1. Hypercoagulability

Under physiological conditions, the endothelium has a crucial role in preserving vascular homeostasis and inhibiting platelet aggregation and coagulation mechanisms [26,27], through the production of endogenous molecules such as NO, prostacyclin, tissue factor inhibitor and thromboxane A_2_ [28].

Consequently, a damaged endothelium inducesvascular contraction and a hypercoagulable state that increases the risk of venous and arterial thromboembolic events [26]. A marker of ED is reduced levels of NO which, underphysiological conditions, induces the synthesis of cyclic guanosine monophosphate (cGMP), with consequential vasodilatory, antioxidative, antimicrobial and antithrombotic actions [29,30]. During viral infection, reactive oxygen species (ROS) increase, recruit M1 macrophages and neutrophils and, in reaction to NO, produce peroxynitrite in order toprevent entry of the virus into host cells. However, this mechanism determines damage to the endothelium, increases vessel permeability, consumption of NO and lipid membrane peroxidation [31]. In the metabolism of NO, ACE and ACE 2 are also involved, and they have opposite roles. ACE stimulates ROS production and inflammation and lessens NO synthesis in addition to vasoconstriction effects [32]. Conversely, ACE2 inactivates Ang II, generated by ACE, and produces Ang (1–7) [32], which is an important NO inducer [29]. As with other viral infections, NO also inhibits SARS-CoV-2 replication [26], but conversely, SARS-CoV-2 infection causes a reduction in ACE2 levels and, consequently, those of its metabolite Ang (1–7), with its predominant action on ACE [33]. This lack of NO stimulation allows viral entry and replication [34]. The reduction in enzymatic activity of ACE2 increases the vascular permeability and expression of tissue factor (TF) in subendothelial cells, as well as leukocytes and platelets. These alterations trigger the cascade of coagulation. Ang II increases the expression of plasminogen activator inhibitor 1 (PAI-1) in endothelial cells, smooth muscle cells and adipocytes, leading to hypofibrinolysis [35]. In this dysfunctional state, the integrity of the endothelium is reduced, and it expresses thromboxane and adhesion receptors, such as those for von Willebrand factor (VWF) and prostacyclin, recruiting platelets, leucocytes [36] and all inflammatory cells, inducing thrombus formation. 

VWF is a coagulation factor that stimulates the adhesion of platelets to the altered endothelium, inflammation and complement activation, and it increases endothelial permeability with the consequential creation of tissue edema [37]. A hyperinflammatory state and viral-specific Toll-like receptors (TLRs) activate the coagulation cascade, leading to platelet aggregation due to increased platelet reactivity. In addition, the activation of neutrophils provokes the release of neutrophil extracellular traps (NETosis), which leads to the adhesion of platelets and red blood cells, which together with the TF pathway increases thrombin and fibrin production, constituting the occlusive thrombus [35]. Increased NETosis is linked with respiratory distress syndrome and is predictive of venous thromboembolism and mortality [38,39]. Activated platelets interact via adhesion proteins such as P-selectin and CD40 with the immune system. In addition, they release α-granules, complement C3 and cytokines such as IL-1β, IL-7, IL-8 and CCL2, activating the immune system [40,41]. In severe COVID-19 patients, platelet-leukocyte aggregates, including monocytes, neutrophils, CD4+ and CD8+ T lymphocytes, has been reported [42,43]. Above all, circulating platelet-monocyte and platelet-neutrophil aggregates correlate with high levels of TF, a trigger for thrombosis [44,45,46].

**Figure 2:**
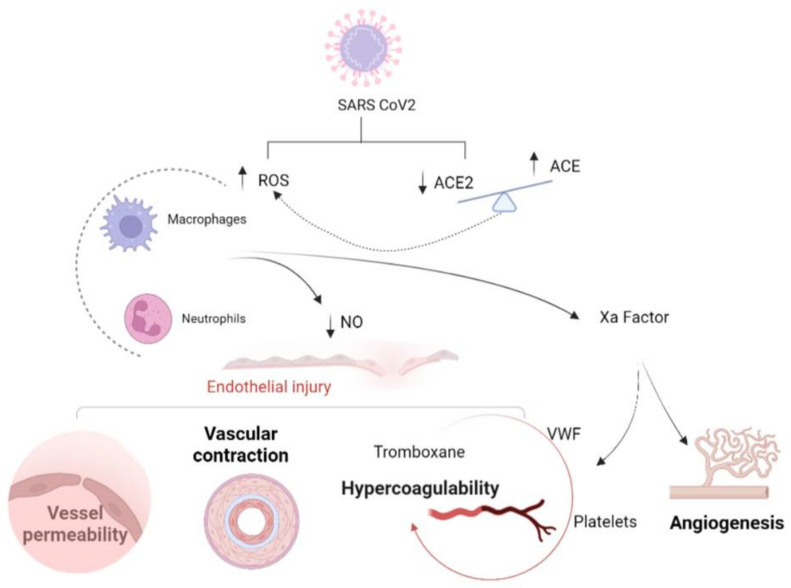
SARS-CoV-2 determines both an increase in ROS and the imbalance of ACE/ACE2. The former mechanism results in recruitment of M1 macrophages and neutrophils, where as the latter, due to the prevalence of ACE, further stimulates ROS production and inflammation. Moreover, it lessens NO synthesis in addition to having a vasoconstriction effect. All these mechanisms induce damage to the endothelium and increase the permeability of vessels, the consumption of NO and lipid membrane peroxidation. Endothelial injury causes increased expression of thromboxane and adhesion receptors, such as those for VWF, recruiting platelets, leucocytes and all inflammatory cells, inducing thrombus formation. This hypercoagulability compromises perfusion of organs, such as the lung, myocardium and kidney, to the point of manifestation of DIC. Furthermore, COVID-19 inflammation activates factor Xa, which stimulates inflammation (through IL-6 induction) and angiogenesis [2,22,26,29,31,32,34,36,37,48,49].

This hypercoagulability compromises the perfusion of organs, such as the lung, myocardium and kidney, to the point of manifestation of DIC (disseminated intravascular coagulation) [26]. Widespread thrombosis can be pointed out by coagulation markers such as elevation of D-dimer, fibrin degradation products and VWF, thrombocytopenia, elevation introponin for reduction of myocardial perfusion [2] and hypoxia for lung dysfunction [47]. Moreover, COVID-19 inflammation activates factor Xa, which is not only the inducer of the extrinsic and intrinsic pathways of coagulation but also stimulates inflammation (through IL-6 induction) and angiogenesis [48] (Figure 2).

Another event that occurs in COVID-19 is, in fact, angiogenesis. There are two types of angiogenesis: sprouting and non-sprouting or intussusceptive (a vessel is divided into two lumens by including circulating angiogenic cells) [48,49,50]. It is induced by several factors such as inflammation, hypoxia(the principal factor) and oxidative stress [51]. Hypoxia, in fact, stimulates the production of some molecules, like hypoxia-inducible factor 1α (HIF-1α), that lead to the synthesis of specific pro-angiogenic factors such as vascular endothelial growth factor (VEGF). Other molecules involved in angiogenesis are VEGFR1, TGF-β, Tie-2 (Ang receptor), cyclooxygenase-2, endothelin, nitric oxide synthase (NOS), AngII, IL-6 and IL-8 [52]. The presence of angiogenesis in COVID-19 has been demonstrated by several autopsy studies conducted onpatients with SARS-CoV-2 infection, onpatients with H1N1 influenza and on normal controls [53]. In COVID-19 patients, autopsies revealed intussusceptive angiogenesis and increased expression of pro-angiogenic factors, such asplatelet-derived growth factor (PDGF), VEGF, VEGFR1, matrix metalloproteinase 2 (MMP-2), TIMP1, HIF-1α, intracellular adhesion molecule1(ICAM-1), super family 1A (TNFRSF1A), IL-6 and ACE2, compared withinfluenza and control patients [53]. Other autopsy studies revealed, in COVID-19 patients, intussusceptive angiogenesis in other organs such asthe heart, liver, kidney, brain and lymphoreticular organs [54]. Moreover, urokinase plasminogen activator(uPA), whichstimulates the transformation of plasminogentoplasmin, induces pro-angiogenic and pro-inflammatory factors [55]. Levels of its receptor (uPAR), in infectiousdiseases, are predictors of an inflammatory state, disease severity, risk of ARDS and mortality [56]. The authors of a previous study supposed that more severe endotheliitis and thrombosis in COVID-19 lungs could induce the intussusceptive angiogenesis observed in these autopsy studies. Although they are preliminary data, it seems that the duration of hospitalization increased the gravity of intussusceptive angiogenesis in COVID-19 patients [53].

### 3.2. Inflammation

Several sets of data from the literature demonstrate that levels of cytokines are elevated in critical patients and that high serum virus RNA levels are combined with high IL-6 levels and with a worse prognosis. In fact, the severe form of SARS-CoV-2 infection is associated with a “cytokine storm” or cytokine-release syndrome (CRS) that can induce multi-organ failure and ARDS. CRS is activated by the NF-Κbsignaling pathway; NF-κB is a transcription factor that is overexpressed by the interaction of SARS-CoV-2 proteins and ACE2. The cytokines involved in this syndrome are IL-6, IL-1β, IL-7 and IL-10 [57]. SARS-CoV-2, indeed, evades the innate immune system, antagonizing the interferon-γ (INFγ) one. This hyperinflammatory state due to elevated levels of tumor necrosis factor (TNF) and IFNγinduces apoptosis and necrosis. The innate immune system cells, like neutrophils and monocytes, located in the nasopharyngeal mucosa secrete chemokines. The major secretion of chemokines is shown in the most severe form of infection. There is also an increase inpro-inflammatory macrophages in the lungs of patients that brings about the activation and differentiation of granulocytes and monocytes. In patients with severe infection, an increase inneutrophils and lower levels of lymphocytes are demonstrated [58].

The other pivotal system is the adaptative immune one, which is important for the progress of infection, and its late response is involved in fatal cases. The cells involved in this system are CD4+, CD8+ and B cells. CD4+ cells induce the recruitment and differentiation of CD8+ and B cells. They also have an important role in anti-pathogen activity, producing specific cytokines. CD8+ cells, instead, destroy the infected cells. Moreover, the production of specific antibodies in the early phases of infection stops the release of the virus from cells. This pro-inflammatory state facilitates endotheliitis by impairment of the anticoagulant processes and inhibition of fibrinolysis, due to the release of PAI-1 [59]. Moreover, the inactivation of ADAMTS-13during sepsis may contribute to the prothrombotic state, due to insufficient cleavage of VWF [60].

These pathophysiological mechanisms of the immune response must be considered because they are responsible for damage that is sometimes more severe than that caused by the virus infection itself. SARS-CoV-2, like other viral infections, has a lot of mechanisms for evading the immune response [61].

All of these mechanisms are connected in a vicious cycle because a cytokine storm damages the endothelium and a dysfunctional endothelium recruits inflammation molecules. So, differences in molecule plasma levels reflect differences in the degree of angiogenesis and in clinical manifestations, which have repercussions on potential treatments and prognosis [62].

## 4. Different Variants of SARS-CoV-2 and Their Impact on Endothelial Dysfunction

During the pandemic, new variants of SARS-CoV-2 developed, resulting in interest in understanding both their clinical implications and the underlying pathophysiological mechanisms. A variant is a virus with some modifications in the genetic code. WHO classified SARS-CoV-2 variants into two types: Variants of concern (VOC) and variants of interest (VOI). The first, comprising Alpha, Beta, Gamma, Delta and Omicron are characterized by increased transmissibility, virulence and resistance to vaccination [63]. The alterations in the protein S, the most variable part of the virus, can facilitate transmission and the elusion of neutralizing antibodies, especially in the Delta and Lambda variants. The foremost reason for their greater pathogenicity is the higher affinity of the S1 domain of the S protein to the ACE2. Moreover, the S protein can activate platelet and coagulation factors, determining thrombosis [64]. The Omicron variant encompassed many changes in the S and RBD region, resulting in a very high-transmission degree and a relatively mild illness severity [65]. Indeed, in a molecular bioinformatics study, the impacts of molecular change between the three significant SARS-CoV-2 variants (Beta, Delta and Omicron) on binding affinity for ACE2 receptor was described, demonstrating that the Omicron variant has the easiest transmission. Mainly, numerous mutations are recognized in the molecular structure of variants that could determine the modification of molecular pathogenesis [66]. Some studies confirmed these data, demonstrating, in addition, that the Omicron group had a decreased severity [67,68]. The underlying hypothesized mechanisms include less efficient infection of lung cells compared with the Delta variant and previous variants and less inhibition of the host cell interferon immune response [69]. McMahan et al. showed that Omicron induced higher viral loads in nasal turbinates than in the lung [70]. These data could be explained by the discovery of recent studies that proposed that Omicron is less specialized in cell tropism, so it infects more types of cells [71]. Moreover, an in vivo study showed that the Omicron variant, when compared with others, affected endothelial cells to a lesser extent [70]. This discovery could, therefore, explain both the reduced incidence of pulmonary embolism associated with this variant [72] and the need to adapt prophylactic therapy with heparin, thus balancing both thrombotic and hemorrhagic risk. In a prospective study conducted on critically ill patients admitted because of COVID-19, Omicron patients had a lower incidence of pulmonary embolism than Delta ones [73]. Finally, Corriero et al. reported that vaccines determine better protection in Omicron patients than Delta ones, and three vaccine doses increase protection in Omicron patients up to 60–75% [73].

## 5. Pulmonary Manifestations and Pathophysiologic Mechanisms

The severity of pulmonary manifestations can be influenced by numerous factors such as viral overload, genetic, ethnicity, comorbidities, age and sex [74]. In this context, COVID-19 can manifest itself in different clinical forms [75]. In fact, pulmonary manifestations can be divided into three degrees: mild, moderate and severe. Mild cases are represented by upper respiratory tract infection (URTI), cough or sore throat [76]. The moderate forms may be characterized by fever and pneumonia that is described as a diffuse interstitial involvement with multiple and bilateral infiltrates, showed by computed tomography imaging as bilateral, asymmetric and prevalent subpleural ground-glass opacities with consolidative pulmonary ones or as crazy paving patterns [77]. A presenting feature of COVID-19 pneumonia is the “silent hypoxia” name, justified by the good tolerability of patients [78]. Lastly, the severe manifestation is ARDS [79], which is an acute onset of hypoxemia with bilateral pulmonary edema related to excessive alveolo–capillary permeability [80]. When SARS-CoV-2 binds to ACE2 receptors, it induces alveolar impairment and interstitial inflammation with macrophage infiltration, formation of hyaline membranes, and alveolar wall edema and thickening. The cells infected by the virus are phagocytosed by dendritic cells and macrophages present in the lung, which triggers the response of T cells, determining the activation of adaptive and innate immune mechanisms [81]. Moreover, the pro-inflammatory stage, mediated by the increase in TNF-α, IL-1β, IL-6 and the aforementioned cytokine storm, plays an important pathophysiologic role [80,82]. Furthermore, in these patients, an immune suppression phase, characterized by lymphopenia, low CD4 and CD8 T cell, increases the risk of bacterial infection [83,84]. In the later disease stages, especially associated with ARDS, a systemic coagulopathy is evident, with widespread microthrombi in the vessels causing pulmonary infarction, hemorrhage, pulmonary hypertension and secondary ventricular injury [85]. In addition, the destruction and damage of the alveolar cells determine a reduction in pulmonary surfactant, leading to an increase in the surface tension of the lung and susceptibility to ARDS [86,87]. Accordingly, pulmonary manifestations are due to alveolar injury, interstitial inflammation, a cytokine storm, immune suppression, diffuse pulmonary intravascular coagulopathy and silent hypoxia [80].

## 6. Extrapulmonary Manifestations and Pathophysiology

Other multiple extrapulmonary manifestations have also been observed, associated with prolonged hospitalization and increased mortality risk. Effectively, extensive SARS-CoV-2 infection–induced endothelial insult and vascular damage are closely linked to severe inflammation, thrombosis and multi-organ failure in critically ill patients. The main mechanisms involved in ED in different organs are summarized in Table 1.

### 6.1. Cardiac Manifestations

Cardiac complications are arrhythmias, acute myocardial injury, myocarditis, cardiomyopathy, thrombosis and myocardial fibrosis. They can be associated with several mechanisms, such as the action of SARS-CoV-2 on ACE2 receptors in the heart with direct viral cytopathic effects [104], adrenergic activation, fluid excess and side effects of treatments [105,106,107]. Likewise, data from the literature document that both a cytokine storm from systemic inflammation and the hypoxic state from ARDS inducing excessive extracellular calcium levels can cause myocyte apoptosis and increase myocardial demand. Moreover, the cytokine-release syndrome depresses myocardial function through the activation of the neural sphingomyelinase pathway [106]. These mechanisms, in the setting of acute infection, can lead to atherosclerotic plaque instability and myocardial injury. The last one predisposes patients to new-onset cardiomyopathy or worsening of pre-existing left ventricular dysfunction [108]. Arrhythmias, moreover, are caused by abnormal signaling from systemic stress or intrinsic cardiac electrical dysregulation. Myocardial damage and hypoxia, due to respiratory failure with the consequent mismatch between oxygen supply and demand [106,108], can lead to electrolyte imbalances, triggering abnormal depolarizations. Atrial arrhythmias are the most frequent; the main ones are sinus tachycardia and atrial fibrillation [109,110].

### 6.2. Renal Manifestations

Common renal abnormalities associated with COVID-19 include proteinuria, hematuria, metabolic acidosis and kidney failure, due to reduced blood flow [115]. Multifactorial processes have been suggested as a cause of kidney damage. First, acute kidney injury is triggered by direct renal cellular harm from SARS-CoV-2 due to ACE2 expression in the renal tubules, data supported by the evidence of acute tubular injury, diffuse erythrocyte aggregation and obstruction in peritubular and glomerular capillary loops [116,117]. Second, the evidence of endothelialitis in the kidney hints that microvascular dysfunction is secondary to endothelial damage [3]. Third, a cytokine storm can induce acute renal failure and disorder of RAAS homeostasis [118,119,120]. Moreover, sepsis causes acute tubular necrosis, multi-organ failure and shock. Among renal manifestations, proteinuria has been mentioned, which is related to ED or direct podocyte injury [117]. SARS-CoV-2 can enter the brain from systemic circulation and through synaptic connections and retrograde neuronal dissemination.

### 6.3. Nervous System Involvement

Nervous system involvement is characterized by ataxia, seizures, neuralgia, unconsciousness, acute cerebrovascular disease, encephalopathy, encephalitis and Guillain–Barré syndrome [121]. These neurological manifestations have been attributed to several mechanisms, such as the virus’s direct effect mediated by the ACE2 receptor distribution in the brain and nerves and the ascent by olfactory nerve axons. Still, ischemic stroke due to viral hypercoagulability, aside from hypoxia that could lead to brain edema and lastly a cytokine storm, affects the brain vasculature and the blood–brain barrier, with damage to the central and peripheral nervous systems [97,98,99,100,101].

Data from the literature demonstrated that isolation and quarantine, over and above the direct effect of COVID-19 itself in the brain tissue, determine the psychological manifestations [102] that include post-traumatic stress, confusion, anger, increased worry, fear, frustration, guilt, isolation, loneliness and nervousness [103].

### 6.4. Gastrointestinal Manifestations

Other significant COVID-19 manifestations include gastrointestinal ones, whose pathophysiological mechanisms are multifactorial. Evidence suggests mainly the direct action of the virus on gastrointestinal epithelium due to widespread expression of ACE2 in intestinal cells [88] and cytokine storms [89,90]. Furthermore, histopathological data onendothelial inflammation in the small intestine vessels and mesenteric ischemia suggest microvascular injury [3]. After all, modification of the intestinal flora induced by the virus could determine gastrointestinal symptoms and severe disease development [91]. Liver injury, characterized by an increase inaminotransferases and bilirubin, and rarely severe acute hepatitis [92,93] are secondary to various mechanisms, such as direct damage of the biliary ducts by bindingofACE2 on cholangiocytes [93], drug-induced hepatotoxicity, antiviral drugs and pneumonia-associated hypoxia [94,96].

### 6.5. Other Manifestations

At last, the inflammation, along with hypoxia and direct viral effects, causes vascular disorders, including arterial thrombotic complications, such as acute limb and mesenteric ischemia, venous thrombotic complications (deep vein thrombosis and pulmonary embolism) and catheter-related thrombosis. The interruption of blood vessel integrity with the consequent activation of platelets that are, moreover, directly triggered by the activation of ACE2 receptors results in platelet hyperactivity and increased thrombus formation. These hemostatic and inflammatory alterations, which reflect endothelial damage, lead to a prothrombotic setting [111,112,113,114].

## 7. Long Terms Consequences

In 2020 the term “long COVID” was created by patients to refer a post-acute condition [122] that is characterized by residual and persistent multi-organ symptoms involving almost two-thirds of COVID-19 recovered patients [123]. The World Health Organization (WHO)’s definition of long COVID is “A condition that occurs in individuals with a history of probable or confirmed SARS-CoV-2 infection, usually three months from the onset of COVID-19 with symptoms that last for at least two months and cannot be explained by an alternative diagnosis [124]”. The exact pathophysiology is unknown [125] but is assumed to be multifactorial. Some hypotheses proposed involve direct viral tissue damage, immune-mediated organ harm [126,127], endothelial injury and hypercoagulability [126].

Long COVID reflects chronic damage to multi-systemic organs [128]. The main pulmonary outcomes are dyspnea, ventilator and/or oxygen requirement, pulmonary function test (PFT) abnormalities and fibrotic lung disease [129,130]. Furthermore, hypercoagulability predisposes the patient to the risk of thrombosis within the small vessels of the pulmonary vasculature [129,131]. Cardiovascular system sequelae include interstitial inflammatory infiltration, myocardial hypertrophy and necrosis [106], as well as myositis, cell death and, finally, fibrosis with the consequent increased risk of arrhythmias. Among cardiac symptoms, palpitations and increased incidences of postural tachycardia syndrome (POTS) have also been described [132]. In addition, the nervous system is also affected by long COVID both with neurodegenerative and thrombotic disorders [133].

Furthermore, COVID survivor patients have complained about neuropsychiatric symptoms, such as chronic malaise, fatigue, sleeping disorder, ageusia and anosmia [134]. Direct and indirect mechanisms could, additionally, damage the other systems such as the kidney, where they can induce focal segmental glomerulosclerosis and glomerular involution [135], as well as induce disorders of metabolic homeostasis [128].

In conclusion, the most frequent lingering symptoms are fatigue, muscle pain, palpitations, cognitive impairment, dyspnea, anxiety, chest pain and arthralgia [129].

## 8. Possible Therapeutic Options

A summary of the most suitable therapeutic approaches used in the treatment of COVID-19-related coagulopathy is reviewed in the following sections.

### 8.1. Role of Heparin

The International Society of Thrombosis and Haemostasis guidelines recommended the administration of low-molecular-weight heparin (LMWH) at a prophylactic dose in all hospitalized COVID-19 patients if there were no contraindications [136].

LMWHs, in addition to antithrombotic activity, exhibit anti-inflammatory activity through inhibition of pro-inflammatory cytokine binding, selectin blockade and inhibition of bradykinin synthesis [137]. Moreover, they have antiviral activity through attenuation of binding between the S protein and the ACE2 receptor [138]. Host cell–virus binding is mediated by docking of the virus S protein with the proteoglycan chain heparan sulfate, which is ubiquitously present on the surface of host cells [139,140] and acts as a coreceptor for viral entry receptor [141,142]. Heparin, having a similar structure to heparan sulfate, acts as a decoy, inhibiting the entry of SARS-CoV-2 into human cells [139]. Several antiplatelet therapy recommendations and protocols have been implemented in the management of patients with COVID-19.

Data from the literature are not unambiguous on the use of heparin at therapeutic or prophylactic doses in hospitalized patients with COVID-19. The INSPIRATION study [143] compared the use of intermediate-dose vs. standard-dose prophylactic anticoagulation among patients with COVID-19 admitted to the intensive care unit (ICU). The study showed that there were no significant differences between the two groups in terms of a reduction in the number of venous or arterial thrombosis events and mortality within 30 days. Hemorrhagic events were more frequent in the group of patients treated with an intermediate dose of enoxaparin. The RAPID study [144] compared the use of therapeutic doses vs. standard doses of heparin in critically ill patients with COVID-19. The study demonstrated a non-significant trend toward a reduction in the number of fatal events at 28 days and in terms of ICU admissions, in the group treated with therapeutic doses of heparin. The risk of all-cause mortality was reduced in the group treated with a therapeutic dose of an anticoagulant. The HEP-COVID study [145] compared therapeutic doses of LMWH vs. prophylactic/intermediate doses of LMWH or unfractionated heparin in critically ill hospitalized patients with COVID-19 and high thromboembolic risk. Therapeutic doses of enoxaparin led to a 32% reduction in the number of venous or arterial thromboembolic events and all-cause mortality. Although no benefit has been found for therapeutic heparin in patients who are critically ill and admitted to ICUs with COVID-19, there is evidence from the literature of a possible role for it, as well as a benefit from the use of therapeutic doses of anticoagulants in high-risk hospitalized patients who do not require ICU support.

Data suggest improved efficacy and safety of the use of weight-optimized prophylactic dosing, at least in non-severe hospitalized patients [146]. There are ongoing randomized clinical trials evaluating the different potencies of heparin anticoagulants, depending on therapeutic or prophylactic dosing [147]. However, according to different institutions, it is good to empirically start therapeutic anticoagulants, in the management of patients with SARS-CoV-2, by performing stratification of the prothrombotic hemorrhagic risk for each individual [139].

### 8.2. Role of Corticosteroids

The use of corticosteroids in respiratory failure related to COVID-19 infection is much discussed. A reduction in thrombotic complications associated with COVID-19-related inflammatory activity has been observed through their anti-inflammatory activity [139] and their ability to induce a reduction in the levels of procoagulant factors such as fibrinogen and VWF [148]. On the other hand, potential risks associated with their use have emerged, such as reduced viral clearance and increased susceptibility to secondary infections [139,149,150,151]. In addition, experimental studies have shown that the use of corticosteroids is associated with increased levels of certain coagulation factors, which could demonstrate how this class of drugs may represent a risk for the development of thrombotic disease [148,152]. Thus, unless there is an indication, such as an exacerbation of chronic obstructive pulmonary disease, there is no proven benefit in the routine use of corticosteroids in the context of patients with COVID-19 [153]. Initially, the WHO advised against the use of glucocorticoids in patients with SARS-CoV-2 [154]; with data from the RECOVERY trial, the therapeutic indications have changed. The trial demonstrated that using dexamethasone at a dose of 6 mg once daily for up to 10 days reduces 28-day mortality only in patients with severe disease that need invasive mechanical ventilation or oxygen therapy [155].

In a recent meta-analysis of clinical trials with1703 patients with severe COVID-19, the administration of systemic corticosteroids was associated with lower 28-day all-cause mortality compared with the usual care or a placebo [156]. Another meta-analysis emphasized that the use of methylprednisolone compared with a placebo, in patients with COVID-19, reduced mortality and the need for mechanical ventilation without increasing the risk of secondary infections but could decrease viral clearance. Moreover, the benefits are greater in cases of severe disease, in which short-term treatment with low-dose methylprednisolone (1–2 mg/kg/day for ≤7 days) is indicated [157].

### 8.3. Role of RAAS Inhibitors

The use of RAAS inhibitors in COVID-19 is controversial because several pieces of evidence emphasize that these drugs can upregulate ACE2 expression and facilitate entry of the virus into cells. On the other hand, RAAS inhibitors could ameliorate ED. ACE2 converts AngII to Ang (1–7), which increases NO release and has an anti-inflammatory and anticoagulant role. BRACECORONA was the first randomized controlled trial that showed that discontinuation of RAAS inhibitors in COVID-19 did not ameliorate mortality risk [158]. A cohort study including 8.3 million people [159] showed that RAAS inhibitors reduce the risk of COVID-19; susceptibility could be associated with the ethnic-specific effects of this drug class. A retrospective observational study and a recent Italian registry demonstrated that RAAS inhibitors do not have deleterious effects on hospitalized people [160,161]. Therefore, there is no evidence for their role to ameliorate ED.

### 8.4. Role of Statins

As well as RAAS inhibitors, statins can also protect the endothelium, through several pathways such as NO, NF-kB and tissue factor expression [28]. Moreover, it is known thatthestatin-mediated reduction inlow-density lipoprotein cholesterol has a positive effect, decreasing oxidative stress and ED [162]. Two meta-analyses showed the beneficial effects of chronic statin use on mortality risk in COVID-19 patients [163,164]. On the other hand, data from RCTs appear controversial, highlighting the safety of statins in these patients but no improvement in outcomes [165]. However, the INSPIRATION study demonstrated a beneficial role of statins when used in the early phases of COVID-19, within 7 days of ICU admission, mediated perhaps by decreasing the inflammatory response [166]. Lee et al. [167] suggested that statins could decrease the effects of COVID-19 in selected patients, but without other evidence, their role in this topic remains uncertain (Table 2).

### 8.5. Role of Antiviral Agents

Remdesivir, a nucleotide analog of adenosine, blocks the replication of viral RNA [168]. Several randomized clinical trials have studied remdesivir; Wang J et al. enrolled 237 hospitalized patients with pneumonia and peripheral oxygen saturation (SpO_2_) < 94% in ambient air. Subjects were randomized 2:1 to receive remdesivir (200 mg intravenous (IV) on the first day and 100 mg from the 2nd day up to a total of 10 days of treatment) or a placebo. The primary endpoint of the study was time to clinical improvement at day 28, but remdesivir did not reach statistical significance [169]. Beigel JH et al. enrolled 1063 subjects, 88.7% with severe disease, that required mechanical ventilation or ECMO (25.6%), non-invasive ventilation or high-flow oxygen (18.5%), oxygen therapy (39.6%) or had mild disease not requiring oxygen therapy (11.9%). Although treatment with remdesivir did not reach statistical significance, a more favorable trend in 14-day mortality was observed in the general population than in the placebo group [170]. In the final report, moreover, a clinical benefit was obtained only in subjects on standard oxygen therapy, while no clinical benefit was observed in subjects on high-flow oxygen therapy, mechanical ventilation or ECMO [170]. Goldman J.D. et al. enrolled 397 subjects, randomized to receive remdesivir for 5 days (n = 200) with severe disease or remdesivir for 10 days (n = 197) with mild–moderate disease. In the group that received remdesivir for 5 days, a numerically lower mortality rate was observed over that seen in the 10-day group (8% vs. 11%), although it was not statistically significant [171]. In the DisCoVeRy trial, adult subjects who were hospitalized with SARS-CoV-2 infection and disease of any duration with pneumonia were treated with remdesivir, but the difference from the placebo group was not statistically significant [172]. In the SOLIDARITY trial, subjects who required mechanical ventilation (both non-invasive and invasive) seemed to have worse clinical outcomes [173]. In the PINETREE trial, authors enrolled non-hospitalized subjects with symptoms within the previous 7 days and who had at least one risk factor for progression to severe COVID-19. Patients were randomized to receive remdesivir (200 mg IV on day 1 and 100 mg on days 2 and 3) or a placebo. The primary endpoint forefficacy was either hospitalization for COVID-19 or death by any cause within the 28th day. The study was suspended early for the reduction of infections, the introduction of vaccines and therapy with monoclonal antibodies. The early therapy with remdesivir reduced hospitalization and death compared with the placebo in patients with a high risk for COVID-19 progression [174].

PF-07321332/ritonavir is composed of two molecules: PF-07321332, a peptidomimetic major protease inhibitor (Mpro) of SARS-CoV-2, also named 3C-like protease (3CLpro) or nsp5 protease, and ritonavir, which inhibits the metabolism of PF-07321332, increasing plasma concentrations. Clinical efficacy is based on the EPIC-HR trial analysis, which included 2245 participants randomized to receive PF-07321332/ritonavir or a placebo orally every 12 h for 5 days. Subjects were non-hospitalized subjects with symptoms within the previous 5 days and who had at least one risk factor for progression to severe COVID-19. The primary endpoint was hospitalization for COVID-19, worsening or death from any cause within 28 days. The incidence of COVID-19-related hospitalization or death by day 28 was lower in the intervention group [175].

Molnupiravir is authorized for the treatment of adults within 5 days of the start of symptoms of COVID-19 that do not require oxygen therapy and are at increased risk of developing severe disease. It is an antiviral that reduces multiplication of SARS-CoV-2. The MOVe-OUT trial enrolled 1734 subjects, randomized to receive molnupiravir or a placebo. Patients were non-hospitalized with the onset of symptoms within 5 days and were at high risk of progression to severe COVID-19. The primary endpoint was time to improvement or resolution of COVID-19 within 29 days. This trial showed the efficacy of early treatment with molnupiravir to reduce hospitalization and deaths in unvaccinated adults with COVID-19 [176] (Table 3).

### 8.6. Role of Monoclonal Antibodies

Monoclonal antibodies act against the S protein of SARS-CoV-2, preventing binding to its receptor. Casirivimab/imdevimab has been evaluated in the clinical trial COV-2067. The primary endpoint was the percentage of patients admitted for COVID-19 or death fromany cause until day 29. It was a double-blind, phase 1–3 trial that included non-hospitalized patients with COVID-19 and risk factors for severe disease. In the intervention group, there was a positive effect on the reduction of the viral load in patients compared with the placebo [177].

Regdanvimab was evaluated in the CT-P59 3.2 trial. Investigators enrolled 1315 adult subjects, who were not hospitalized and with at least one or more symptoms of COVID-19 for 7 days, oxygen saturation > 94% in ambient air and without a need for additional oxygen therapy, randomized to receive a single infusion of regdanvimab at a dose of 40 mg/kg (N = 656) or the placebo (N = 659) over60 min. The primary endpoint was hospitalization or a need for oxygen therapy or mortality onthe 28th day. In patients with mild-to-moderate COVID-19, this monoclonal antibody showed a reduction in hospitalization and oxygen therapy [178].

The efficacy of sotrovimab was evaluated in the COMET-ICE trial, which included 1057 adult non-hospitalized subjects with the onset of symptoms of COVID-19 ≤ 5 days after and at least one risk factor for severe disease, randomized 1:1 to receive a single infusion of sotrovimab at a dose of 500 mg or the placebo. The primary endpoint was hospitalization or death within 29 days. This trial showed that early treatment with sotrovimab reduced the progression of COVID-19 [179].

A major study, the PROVENT trial, which included over 5000 subjects, showed that treatment with tixagevimab-cilgavimab reduced the risk of infection with COVID-19 by 77% in pre-exposure prophylaxis. However, laboratory studies have shown that the Omicron BA.1 variant appears to be less sensitive than the Omicron BA.2 variant at 150 mg tixagevimab and cilgavimab [180] (Table 4).

## 9. Conclusions

In conclusion, ED in COVID-19 can be summarized by four alterations: 1. apoptosis of ECs with loss of integrity, augmented permeability and edema formation; 2. production of adhesion molecules and inflammatory cytokines that recruit inflammatory cells; 3. metabolic alteration; and 4. de-differentiation with consequent angiogenesis [2]. ED plays an important role in severe disease, and it appears to be induced directly by SARS-CoV-2 infection and by systemic inflammation [22] with a dysregulated immune response, which leads to a vicious cycle between ED and a cytokine storm [75]. Literature data confirm that COVID-19increases the release of coagulation factors and dysregulation and destruction of ECs, enhancing the risk of thromboembolic events [181], worsening by older age, immobilization, invasive mechanical ventilation and central venous catheterization [114].

## Figures and Tables

**Table 1:** Clinical manifestations of SARS-CoV-2 infection and pathophysiology.

Organ or System Involved	Clinical Presentation/Disease	Laboratory/Exams	Pathophysiology
Lung [74,75,76,77,78,79,80,81,82,83,84,85,86,87]	Cough (mostly dry), dyspnea, fatigue, sore throat, rhinorrhea, sneezing and, in severe cases, pneumonia, respiratory failure or acute respiratory distress syndrome (ARDS)	Moderate/severe hypoxemia, ground-glass opacities in chest X-rays	Alveolar injury, interstitial inflammation, cytokine storm, immune suppression, diffuse pulmonary, intravascular coagulopathy, silent hypoxia
Gastrointestinal [88,89,90,91,92,93,94,95,96]	Nausea, vomiting, diarrhea, heartburn, loss of appetite, abdominal pain andbloating	Elevated liver enzymes andbilirubin	Direct action of virus on the ACE2 receptors, drugs hepatotoxicity, pneumonia-associated hypoxia
Brain [97,98,99,100,101]	Hyposmia–anosmia, hypogeusia–ageusia, visual disturbance, fatigue,somnolence, headaches, nausea and vomiting, dizziness, myalgia, ataxia, encephalopathy, cerebrovascular disease (large vessel strokes), seizures, meningoencephalitis, neuropathy, Guillain–Barrésyndrome, neurogenic ARDS, coma	Elevated creatine kinase withmyalgia, brain MRI showing hyperintensities in regions with infarction or encephalitis, SARS-CoV-2 detection in cerebrospinal fluid or brain tissues in some patients	Virus’s direct effects mediated by the ACE2 receptors, the ascent by olfactory nerve axons, ischemic stroke due to viral hypercoagulability, hypoxia, cytokine storm
Mental/Psychiatric [102,103]	Post-traumatic stress disorder, depression, anxiety, insomnia, anger, fear, exacerbation ofneurological or psychiatric disorders	Elevated plasma calcium andphosphorus (indicative of stress)	Isolation and quarantine, the effect of COVID- 19 in the brain tissue
Heart [104,105,106,107,108,109,110]	Chest pain, heart attack, myocardial injury, arrhythmias, cardiogenic shock and even sudden death	Elevated cardiac enzymes, abnormal EKG (prolonged QTc intervals, elevated ST), cardiac-specific troponin and brain natriuretic peptide	The effect of SARS-CoV-2 on the ACE2 receptors, adrenergic activation, electrolytic imbalances, fluid excess, side effects of treatments, cytokine-release syndrome, hypoxia
Blood vessels [111,112,113,114]	Pulmonary embolism, disseminated intravascular coagulation, deep vein thrombosis, large vessel stroke, systemic arterial and venous thromboembolism	Elevated D-dimer, interleukin-6, other cytokines, ferritin and lactate dehydrogenase, prolonged PT/PTT	Direct effect of SARS-CoV-2, Platelet hyperactivity and increased thrombus formation
Kidney [115,116,117,118,119,120,121]	Renal failure, tubular necrosis	Proteinuria, hematuria	Direct viral injury, Cytokine storm, Disorder of renin–angiotensin– aldosterone system (RAAS) homeostasis

**Table 2:** Role of different therapeutic approaches in COVID-19.

Therapeutic Approach	Mechanism	Conclusions from the Literature
Heparin	-antithrombotic activity [137] -antiviral activity [138] -inhibits the entry of SARS-CoV-2 into human cells [139]	The INSPIRATION study showed that there are no significant differences between the use of intermediate-dose vs. standard-dose prophylactic anticoagulants among patients with COVID-19 in terms of reduction invenous or arterial thrombosis [143]. The RAPID study demonstrated a non-significant trend toward a reduction in the number of fatal events [144]. The HEP-COVID study [145] showed a 32% reduction in the number of venous or arterial thromboembolic events and all-cause mortality. Data suggest improved efficacy and safety of the use of weight-optimized prophylactic dosing in non-severe hospitalized patients [146].
Corticosteroids	-anti-inflammatory activity [139] -reduction in the levels of procoagulant factors such as fibrinogen and VWF [148]	The RECOVERY trial demonstrated that using dexamethasone reduces 28-day mortality only in patients with severe disease [155]. The benefits are greater in cases of severe disease, in which short-term treatment with low-dose methylprednisolone is indicated [157].
RAAS Inhibitors	-upregulate ACE2 expression that converts AngII to Ang and increases NO release with an anti-inflammatory and anticoagulant role [158]	A cohort study showed that RAAS inhibitors reduce the risk of COVID-19 [159]. There is no evidence for their role to ameliorate ED.
Statins	-reduce low-density lipoprotein cholesterol and oxidative stress [162]	The INSPIRATION study demonstrated the beneficial role of statins by decreasing the inflammatory response [166].

**Table 3:** Role of antiviral agents in COVID-19.

Antiviral Agents	Mechanism	Conclusions from the Literature
Remdesivir	Blocks the replication of viral RNA [168]	Clinical benefit was obtained only in subjects on standard oxygen therapy, while no clinical benefit was observed in subjects onhigh-flow oxygen therapy or on mechanical ventilation or ECMO [170]. The early therapy (from 3 to 5 days) with remdesivir reduced hospitalization and deaths compared with theplacebo in patients with a high risk for COVID-19 progression [174].
PF-07321332/ritonavir	PF-07321332 is a peptidomimetic major protease inhibitor (Mpro) of SARS-CoV-2 and ritonavir is an inhibitor of the metabolism of PF-07321332 [175]	Treatment within 5 days ofthe start of symptoms, in subjects who were non-hospitalized and with at least one risk factor for progression to severe COVID-19, reduced hospitalization for COVID-19, worsening or deaths from any cause within 28 days[175].
Molnupiravir	Reduces multiplication of SARS-CoV-2 [176]	Early treatment (within 5 days of the start of symptoms) with molnupiravir showed reduced hospitalization and deaths in unvaccinated adults with COVID-19 [176].

**Table 4:** Role of monoclonal antibody in COVID-19.

Monoclonal Antibodies	Mechanism	Conclusions from the Literature
Casirivimab/imdevimab	Two neutralizing immunoglobulin gamma 1 (IgG1) human monoclonal antibodiesagainst the spike protein SARS-CoV-2 [177].	A single intravenous or subcutaneous dose within 3 days of the start of SARS-CoV-2 infection reduced hospitalizations, deaths and viral load in non-hospitalized adults with COVID-19; the treatment improved survival and reduced the risk of worsening or death in hospitalized patients with severe COVID-19 [177].
Regdanvimab	Blocks the interaction regions between SARS-CoV-2 S protein RBD and ACE2 [178].	A single infusion showed a trend toward a minor decrease in time to negative conversion of RT-qPCR results and reduced the need for hospitalization and oxygen therapy in patients with mild-to-moderate COVID-19 [178].
Sotrovimab	Neutralizes SARS-CoV-2 directly targeting the SARS-CoV-2 spike glycoprotein [179].	A single infusion within 5 days of the onset of symptoms reduced hospitalizations, deathsand admissions to ICU in high-risk non-hospitalized patients with COVID-19 [179].
Tixagevimab-cilgavimab	Bind epitopes of the SARS-CoV-2 spike-protein receptor-binding domain to neutralize the virus [180].	A single dose reduced infection and severe disease in people who had an increased risk of an inadequate response to COVID-19 vaccination, an increased risk of exposure to SARS-CoV-2 or both. It was ineffective against Omicron BA.1, whereas it was effective against BA.2 [180].

## Data Availability

Not applicable.

## Abbreviations

3CLpro	3C-like protease
ACE2	angiotensin-converting enzyme 2
ADAM17	ADAM metallopeptidase domain 17
Ang	angiotensin
ARDS	acute respiratory distress syndrome
cGMP	cyclic guanosine monophosphate
COVID-19	coronavirus disease 2019
CRS	cytokine-release syndrome
DIC	disseminated intravascular coagulation
E	envelope
ECs	endothelial cells
ED	endothelial dysfunction
HIF-1α	hypoxia-inducible factor 1α
ICAM-1	intracellular adhesion molecule 1
ICU	intensive care unit
INFγ IV	Interferon-γ intravenous
LMWH	low-molecular-weight heparin
M	membrane-associated
MMP-2	matrix metalloproteinase 2
Mpro	major protease inhibitor
N	nucleocapsid
NETosis	neutrophil extracellular traps
NF-κB	nuclear factor κB
NO	nitric oxide
PAI-1	plasminogen activator inhibitor 1
PDGF	platelet-derived growth factor
PFT	pulmonary function test
POTS	postural tachycardia syndrome
RAAS	renin–angiotensin–aldosterone system
RBD	receptor-binding domain
ROS	reactive oxygen species
S	spike
SARS-CoV-2	Severe acute respiratory syndrome coronavirus 2
SDC1	syndecan-1
SpO2	peripheral oxygen saturation
sVCAM1	soluble vascular cell adhesion molecule 1
TF	tissue factor
TLRs	Toll-like receptors
TMPRSS-2	transmembrane protease serine protease-2
TNF	tumor necrosis factor
uPA	urokinase plasminogen activator
URTI	upper respiratory tract infection
VEGF	vascular endothelial growth factor
VWF	von Willebrand factor
WHO	World Health Organization
